# Limitations of Quantitative Blush Evaluator (QuBE) as myocardial perfusion assessment method on digital coronary angiograms

**Published:** 2018-07-02

**Authors:** Haryadi Prasetya, Marcel A.M. Beijk, Praneeta R. Konduri, Thabiso Epema, Alexander Hirsch, Pim van der Harst, Ed van Bavel, Bas A.J.M. de Mol, Henk A. Marquering

**Affiliations:** ^1^Department of Biomedical Engineering Physics, Academic Medical Center, Amsterdam, the Netherlands; ^2^Department of Cardio-thoracic Surgery, Academic Medical Center, Amsterdam, the Netherlands; ^3^Department of Cardiology, Academic Medical Center, Amsterdam, the Netherlands; ^4^Department of Radiology and Nuclear Medicine, Academic Medical Center, Amsterdam, the Netherlands; ^5^Center for Experimental and Molecular Medicine, Academic Medical Center, Amsterdam, the Netherlands; ^6^Department of Cardiology and Thorax Surgery, University Medical Center Groningen, Groningen, the Netherlands

**Keywords:** myocardial perfusion, myocardial infarction, quatitative blush evaluator, coronary angiogram

## Abstract

**Background and Aim:**

Quantitative Blush Evaluator (QuBE) is a software application that allows quantifying myocardial perfusion in coronary angiograms after a percutaneous coronary intervention. QuBE has some limitations such as the application of a crude filter to remove large scale structures and the absence of correction for cardiac motion. This study investigates the extent of these limitations and we hypothesize that enhanced image analysis methods can provide improvements.

**Methods:**

We calculated QuBE scores of 117 patients from the HEBE Trial and determined its association with the Myocardial Blush Grade (MBG) score. Accuracy of large-structure removal is qualitatively assessed for various sizes of a median filter. The influence of cardiac motion was evaluated by comparing the blush curve and QuBE score of the native QuBE with manually motion-corrected QuBE for 40 patients. The effect of different kernel sizes and motion correction to a potential improvement of the association between QuBE score and MBG was studied.

**Results:**

In our population, there was no significant association between QuBE score and MBG (*p* = 0.14). Median filters of various kernel sizes were unable to remove large structure related noise. Variations in filters and cardiac movement correction did not result in an improvement in the association with MBG scores (observer 1: *p* = 0.66; observer 2: *p* = 0.72).

**Conclusions:**

There was no significant association of QuBE with MBG scores in our population, which suggests that QuBE is not suitable for a quantitative assessment of myocardial perfusion. Alternative kernel sizes for the large structure removal filter and cardiac motion correction did not improve QuBE performance.

**Relevance for patients:**

Further improvements of QuBE to overcome its inherent limitations are necessary in order to establish QuBE as a reliable myocardial perfusion assessment method.

## 1. Introduction

Myocardial infarction is commonly treated by primary percutaneous coronary intervention (PCI) in which various procedures such as coronary angioplasty, stent placement, or thrombus aspiration are performed. PCI aims to reestablish epicardial blood flow in the infarct-related artery and myocardial perfusion. After successful PCI, myocardial perfusion can be assessed using angiography in order to determine if the restored epicardial patency also leads to proper perfusion in the infarcted area [1,2]. The Myocardial Blush Grade (MBG) is one of the most common reperfusion scales for categorization of the quality of perfusion in this area [1]. Although MBG has been proven to be a strong predictor of mortality in patients with restored epicardial flow as indicated by Thrombolysis in Myocardial Infarction flow grade 3, it is a rather coarse scale and is also sensitive to observer dependency. This has prompted the need for an automated and quantitative approach for assessing myocardial perfusion.

Currently, quantification of myocardial perfusion is possible with Single Photon Emission Computed Tomography, Positron Emission Tomography, Cardiovascular Magnetic Resonance, and CT imaging [3–5]. However, these methods require other imaging modalities in addition to the current standard practice of using x-ray angiography during PCI. Therefore, Quantitative Blush Evaluator (QuBE) has been introduced to semi-quantitatively assess myocardial perfusion from coronary angiograms [6].

QuBE is an open-source computer program, which has been developed by the University Medical Center Groningen, the Netherlands [6]. In general, angiographic quantification of myocardial blush poses some difficulties including cumbersome assessment because of poor blush signal to noise ratio and superim-position of irrelevant structures. Recognizing and solving these issues are important in developing a blush quantification method such as QuBE. QuBE has been validated as a good risk predictor in the TAPAS trial, which was a study that included patients with PCI and in which the MBG score was assessed on angiograms. In this study, high QuBE values were associated with high MBG scores, more ST-segment elevation resolution, smaller infarct size, and lower 1-year mortality rate [6]. Although QuBE has been shown to be reproducible, unknown effects of different angiography hardware and techniques, median filter insufficiency as the default large structure removal method, and uncalibrated scoring remain as limitations [7–9]. These inherent limitations might obstruct accurate calculation of myocardial blush. Another possible limitation is the effect of cardiac motion on QuBE score calculation, which has not been studied before. In this study, we evaluate the accuracy of QuBE in a clinical trial data and analyze whether general difficulties of blush quantification and inherent limitations of QuBE can be resolved with enhanced image analysis methods.

## 2. Materials and Methods

2.1 Patients

We included patients with ST-segment elevation myocardial infarction who underwent primary PCI in the HEBE trial [10]. The HEBE trial was a multi-center randomized trial with blinded evaluation of endpoints. This trial was designed to assess the effects of intracoronary infusion of bone marrow mononuclear cells and peripheral blood mononuclear cells in improving left ventricular recovery after acute myocardial infarction. Patients from the bone marrow mononuclear cells, peripheral blood, and control groups were included based on the following criteria: age 30-75 years old, successful PCI within 12h after onset of symptoms, >3 hypokinetic or akinetic left ventricular segments observed on echocardiography at least 12h after PCI, and an elevation of creatine kinase in venous blood >10 times the local upper limit of normal. In addition, patients with hemodynamic instability, upcoming additional PCI, coronary-artery bypass grafting within the next 4 months, severe comorbidity, and contraindications for MRI were excluded from this trial. We included patients from the two largest of the eight participating centers in this study. We included 58 patients from the Academic Medical Center and 87 patients from the University Medical Center Groningen. 14 Coronary angiograms made during primary PCI were collected. The inclusion criteria for accepted angiogram adhered to the guideline provided in the initial study of QuBE [6]. We included complete blush sequence and no major overlapping of other non-infarct related area in myocardial region of interest.

2.2. QuBE evaluations and myocardial blush grade

**Figure 1:**
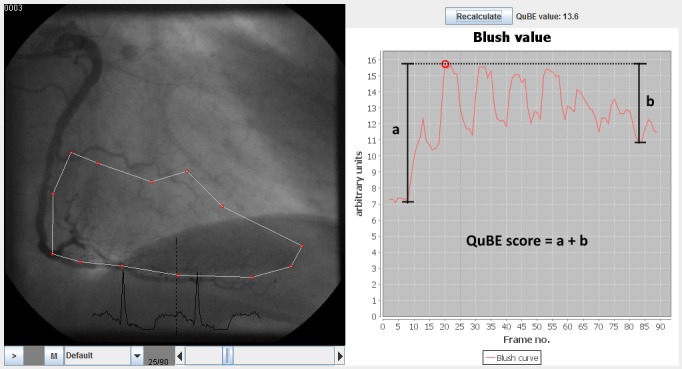
Left: Coronary angiogram with a ROI containing distal infarctrelated area of right coronary artery. Right: Blush curve representing the average intensity of ROI for each frame. The QuBE score is defined as the sum of the maximum increase (a) and the maximum decrease (b) of intensity.

In coronary angiograms, tissue perfusion appears as a blush surrounding the coronary artery. Therefore, myocardial perfusion can be observed by monitoring the dynamics of average contrast intensity within a certain region of interest (ROI), which is shown as a typical curve in Figure 1.

**Figure 2:**
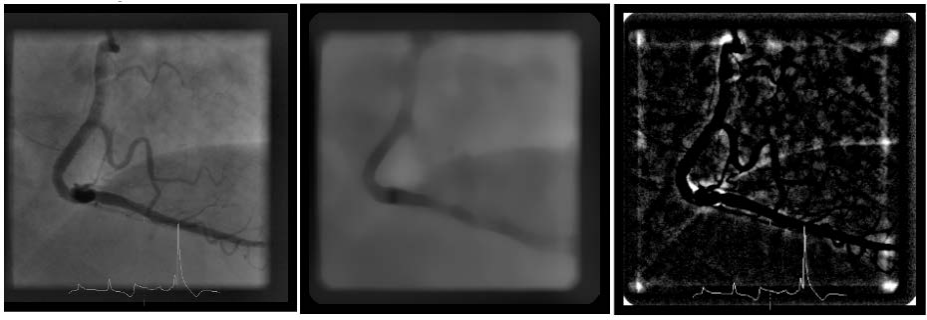
Large structure removal for blush extraction. The original frame of coronary angiogram (left) was filtered using median filter with kernel size of 35 pixels × 35 pixels. The resulting background (middle) was subtracted from the original image such that only blush and other smaller structures remain (right).

The accuracy of QuBE score calculation assumes that the blush can be isolated by removal of contributions from coronary arteries and background structures such as the diaphragm and catheter from the image using filters. This implemented removal of these structures is based on differences between the spatial frequencies of myocardial blush compared to the unwanted structures (Figure 2). QuBE applies a median filter, which creates an image depicting large-scale structures only [11]. Subsequently, this background image is subtracted from the original frame.

This process results in an image representing myocardial blush and other high-spatial frequency noise. The noise characteristics, such as the sparsity and the intensity, depend on the kernel size of the median filter.

The native QuBE software uses a fixed kernel size of 35 pixels × 35 pixels. We evaluated the appropriateness of this kernel size for removal of large structures by comparing with results obtained from two different kernel sizes: 20 pixels × 20 pixels and 50 pixels × 50 pixels. The performance of median filters with different kernel sizes was qualitatively and quantitatively assessed.

Since QuBE uses a fixed ROI location, a bias may be introduced due to the cardiac motion. The QuBE only includes a rudimentary panning motion correction by calculating a possible translation offset of every frame, while cardiac motion is a complex combination of translation, rotation, and non-isotropic contraction and relaxation. We evaluated whether additional cardiac motion correction improves the agreement of QuBE score with MBG. The comparison was made because MBG is the most commonly used angiographic measure to assess myocardial perfusion and has moderate to good inter- and intra-observer agreement [1,12,13]. For this, a single experienced cardiologist who was blinded to clinical data first indicated the ROI on a frame of reference. Two trained observers subsequently manually adjusted the ROIs for all time frames, ensuring that the ROI indicates the same area of myocardium at all times. The cardiac motion correction was performed for 40 patients (10 of each MBG group).

The suitability of the angiographic angulation was assessed by an experienced cardiologist to avoid an overlap between infarcted and healthy myocardium. The right anterior oblique view of *−*30° and the left anterior oblique view of -60° to *−*90° were considered to be the appropriate angulations for perfusion assessment for the left anterior descending artery. A deviation of ±10° from the two proposed projections was allowed. In appropriate angiograms, the MBG was assessed by the same cardiologist. The cardiologist delineated the ROI that contained the distal part of the perfusion area of the infarct-related artery. The MBG was scored based on the following classification: MBG 0 for no myocardial blush, MBG 1 for minimal myocardial blush, MBG 2 for moderate myocardial blush but less than that obtained during angiography of the reference artery, and MBG 3 for normal myocardial blush that is comparable to the angiographically healthy reference artery.

2.3. Statistical analysis

QuBE scores were summarized as medians (interquartile range, IQR). Associations between QuBE scores and MBG grades were analyzed by calculating the Spearman rank correlation coefficients. Kruskal-Wallis tests were performed to analyze the differences in QuBE scores between MBG groups. Lin’s concordance coefficient was calculated to quantify interobserver agreement on the QuBE scores acquired after manually correcting the cardiac motion. The significance of the difference of the QuBE scores with and without cardiac motion on QuBE score was analyzed using Wilcoxon signed-rank test. The similarity of the native and motion-corrected blush curves was analyzed using Pearson correlation where the intensities for every time frame was compared for both assessments. P-values lower than 0.05 were considered statistically significant. All statistics were performed using IBM SPSS software (version 19.0.0).

## 3. Results

**Figure 3:**
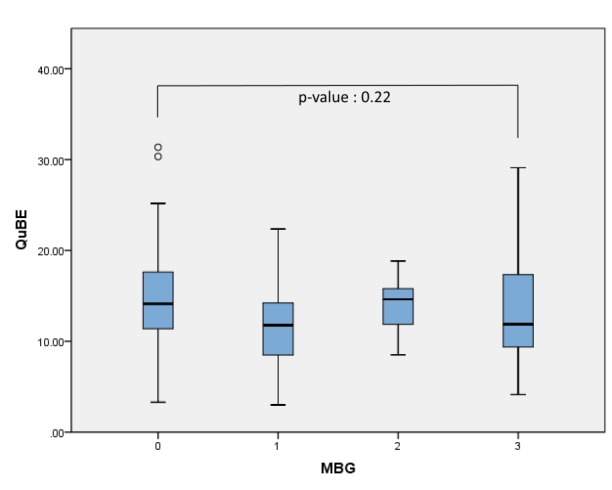
Association of myocardial blush grade with QuBE. MBG 0: no myocardial blush; MBG 1 : minimal myocardial blush; MBG 2: moderate myocardial blush; MBG 3 : normal myocardial blush.

Out of 145 patients, 28 were excluded due to an unsuitable angulation. The remaining 117 patients (48 patients from the Academic Medical Center and 69 from the University Medical Center Groningen) were included in this analysis. The QuBE score distribution for the MBG grades are represented in Figure 3. The correlation between QuBE score and MBG was not significant (*p* = 0.14) and no significant differences were found between the grades (*p* = 0.22). Table 1 summarizes the QuBE scores stratified for MBG scores for varying kernel sizes of the median filter.

**Table 1:** MBG and QuBE score of 117 patients

		MBG 0	MBG 1	MBG 2	MBG 3
n		70	14	13	20
QuBE score
	Kernel Size 20×20	4.2(1.1-2.4)	4.0(1.4-9.4)	4.7(2.3-8.6)	4.9(2.4-9.0)
	Kernel Size 35×35 (Native)	14(3.3-31)	12(3.0-22)	15(8.5-19)	12(4.1-29)
Kernel Size 50×50	15(4.0-35)	12(5.0-22)	15(7.9-18)	13(4.7-36)

**Figure 4:**
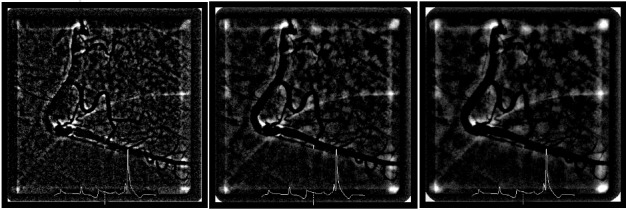
The remaining structure after the median-filtered frame is subtracted from the original frame. Left: kernel size of 20×20. Middle: kernel size of 35×35 (native QuBE). Right: kernel size of 50×50. Contrast is readjusted for clarity.

Figure 4 shows the resulting images after subtracting median filtered images for various sizes of the median filters for a single patient. For all kernel sizes the right coronary and right marginal artery were successfully removed. However, the resulting images were commonly noisy, especially around the edge of the angiogram’s border, arteries, and diaphragm. This figure indicates that a kernel size of 20×20 resulted in more pronounced and higher frequency noise. On the other hand, a kernel size of 50×50 resulted in a lower noise level but in larger areas around the edges of large structures.

**Figure 5:**
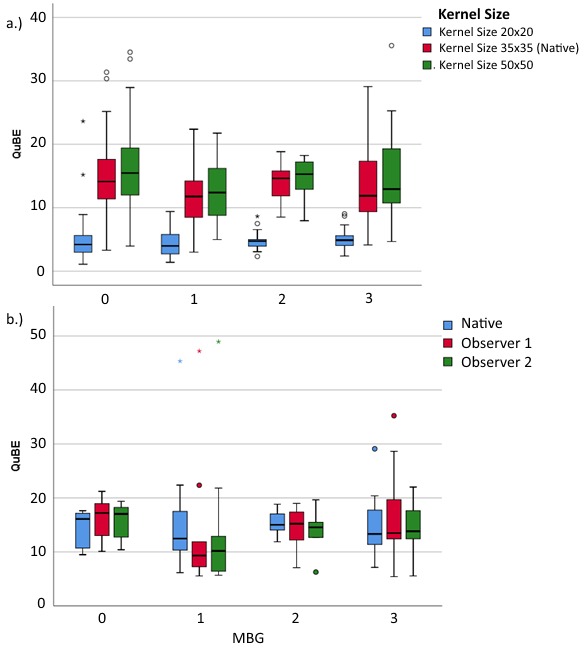
QuBE scores distribution per MBG: (a) for three different kernel sizes of median filter, and (b) pre- and post-motion correction in 40 patients.

Figure 5a shows the distribution of the QuBE scores for varying kernel size and MBG score. We found that there were no significant correlations between QuBE score and MBG for kernel size 20 pixels x 20 pixels (p= 0.33) and 50 pixels x 50 pixels (p= 0.16). Additionally, no significant QuBE differences were found between MBG groups for all kernel sizes (p= 0.70 and 0.28 for kernel size 20 pixels x 20 pixels and 50 pixels x 50pixels, respectively).

**Figure 6:**
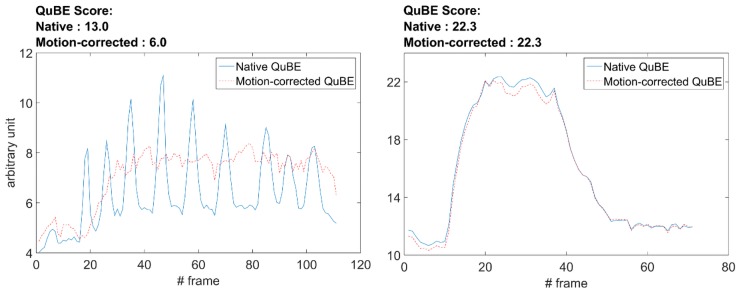
Comparison of the native QuBE and motion-corrected QuBE of blush curves. The frame rate is 12.5 frames per second. The largest difference in blush curves is shown in the left panel (*R* = 0.47). The right panel shows the best correlation between the two blush curves (*R* = 1.00).

There were no significant differences among QuBE scores of different MBG groups of the native and motion-corrected QuBE score (p = 0.70), as can be seen in Figure 5b. For both observers, 38 patients demonstrated strong correlation between blush curves of native and motion-corrected QuBE and the remaining 2 patients showed moderate correlation (observer 1: median *R* = 0.97, range 0.47-1.00; observer 2: median *R* = 0.98, range 0.53-1.00). The Lin’s inter-observer concordance was 90%. The native and motion corrected blush curves with the worst and the best correlation are shown in Figure 6. The Wilcoxon signed-rank test showed that additional manual cardiac motion correction performed by the two observers did not elicit a statistically significant change in QuBE scores (*p* = 0.66, 0.72).

## 4. Discussion

In our population, we found no association between QuBE scores and the MBG score, suggesting that QuBE is not suitable for myocardial blush quantification. We found that the implemented median filter is not accurate in the removal of large structures and that in the filtered images many artefacts associated with large structures remain and influence the QuBE score. We explored different sizes of filters without better results. Furthermore, cardiac motion correction did not strongly affect QuBE calculation. These findings suggest that despite the reported high reproducibility, QuBE scores may not represent the actual reperfusion state.

The feasibility of QuBE has been evaluated in a number of trials, notably the TAPAS and PREPARE trials [8,9,14,15]. These authors found that a high QuBE score significantly correlates with high MBG, ST-segment elevation resolution, smaller infarct sizes, survival at 1 year, improved functional outcome, and contrast-enhanced Cardiac Magnetic Resonance outcomes [6,8,9]. Our results do not confirm these findings.

Because QuBE is open source, it allowed for detailed inspection of the algorithms that are employed in the software. We found that the underlying cause of the lack of association between QuBE and MBG may reside within QuBE itself. We have shown that the median filter used in QuBE may not be appropriate for blush isolation. It was demonstrated that the filtered image may contain noise around the edges of removed structures that has the same spatial characteristics as the blush. QuBE calculates the local average of the intensities of the few brightest pixels as the blush value of a single frame of angiogram [6]. This calculation leads to the inclusion of the noise in the equation since there is no earlier process in QuBE that distinguishes blush from the noise.

We considered cardiac motion as a potential important limitation in the calculation of the QuBE score. Our observation, however, revealed that in most cases cardiac motion did not have a large influence on the QuBE calculation. We suspect that the limited improvement of cardiac motion correction is because the ROIs were large enough for the infarct-related artery and its perfusion area to remain inside the ROI during the cardiac cycle. On the other hand, in the cases where the ROI is close to a coronary artery bifurcation but does not include it, i.e., during reperfusion assessment of myocardium supplied by the right coronary artery, cardiac motion did have an effect. Since the most prominent cluster of noise was formed in curving arteries and bifurcations, the cardiac motion which subsequently included and excluded this bifurcation in a cardiac cycle introduced subsequent spikes and dips in the blush curve. In these particular cases, motion correction may improve the accuracy of the QuBE score.

Describing and visualizing intermediate results in QuBE calculations set this study apart from previous QuBE studies. This allowed for careful analysis of the limitations of the specific algorithms in QuBE. Although we investigated different kernel sizes of the filter, we did not explore other large-scale structure removal methods that might provide better isolation of the myocardial blush. Several enhanced-image and segmentation methods could be employed as alternatives to median filter, i.e., digital subtraction angiography for coronary arteries or vesselness filters for better artery removal [16,17]. Since this is a retrospective analysis of trial data, no power analysis and sample size calculation were performed. Uneven distribution of samples across MBG groups may have reduced the statistical power of our findings. Additionally, the trial data used by previous studies that showed positive findings with QuBE were not available, thus, a comparison study could not be performed. However, aside from the particular limitation of the local algorithm, this discrepancy of QuBE performance may also have been caused by a number of other factors. For instance, type and volume of contrast agent, speed of injection, and the configuration of acquisition machine have not been yet standardized. Besides, the infarct location and body mass index has been known to confound QuBE value [7]. If the image acquisition protocol is standardized and the known confounders are controlled, QuBE may give a more reliable assessment. This information should be incorporated in the guidelines on the use of QuBE to assess myocardial perfusion.

In summary, QuBE may not reliably describe myocardial perfusion and extensive motion correction does not improve its performance. Alternatives for the currently used large-scale structure removal algorithms should be investigated.
